# Controlled Lighting and Illumination-Independent Target Detection for Real-Time Cost-Efficient Applications. The Case Study of Sweet Pepper Robotic Harvesting

**DOI:** 10.3390/s19061390

**Published:** 2019-03-21

**Authors:** Boaz Arad, Polina Kurtser, Ehud Barnea, Ben Harel, Yael Edan, Ohad Ben-Shahar

**Affiliations:** 1 Department of Computer Science, Ben-Gurion University of the Negev, Beer-Sheva 8410501, Israel; ehud.barnea@gmail.com (E.B.); ben-shahar@cs.bgu.ac.il (O.B.-S.); 2 Department of Industrial Engineering and Management, Ben-Gurion University of the Negev, Beer-Sheva 8410501, Israel; benhare@post.bgu.ac.il (B.H.); yael@bgu.ac.il (Y.E.)

**Keywords:** Flash-No-Flash, outdoor vision, fruit detection, autonomous harvesting

## Abstract

Current harvesting robots are limited by low detection rates due to the unstructured and dynamic nature of both the objects and the environment. State-of-the-art algorithms include color- and texture-based detection, which are highly sensitive to the illumination conditions. Deep learning algorithms promise robustness at the cost of significant computational resources and the requirement for intensive databases. In this paper we present a Flash-No-Flash (FNF) controlled illumination acquisition protocol that frees the system from most ambient illumination effects and facilitates robust target detection while using only modest computational resources and no supervised training. The approach relies on the simultaneous acquisition of two images—with/without strong artificial lighting (“Flash”/“no-Flash”). The difference between these images represents the appearance of the target scene as if only the artificial light was present, allowing a tight control over ambient light for color-based detection. A performance evaluation database was acquired in greenhouse conditions using an eye-in-hand RGB camera mounted on a robotic manipulator. The database includes 156 scenes with 468 images containing a total of 344 yellow sweet peppers. Performance of both color blob and deep-learning detection algorithms are compared on Flash-only and FNF images. The collected database is made public.

## 1. Introduction

Commercialization of precision harvesting robots continues to be a slow and difficult process due to the vast challenges in outdoor agricultural and horticultural environments. A major limitation is the low detection rate [1], caused by the unstructured and dynamic nature of both the objects and the environment [2,3,4,5]: fruits have a high inherent variability in size, shape, texture, and location; a typical scene is highly occluded, and variable illumination conditions (caused by changing sun direction, weather conditions, and artificial shades and natural objects) significantly influence detection performance.

Significant R&D has been conducted on detection for agricultural robots [1,3,6]. A short summary of previous relevant results in these reviews and several additional relevant papers is available in Table 1.

The lack of data and ground truth information [13] in the agricultural domain is a major challenge that current, most-advanced algorithms face due to the need for major datasets to be collected (such as the DeepFruits dataset [4]). Best detection results are achieved for crops with a high fruit to image ratio (e.g., apples, oranges, and mangos that grow in high density). Some research [9,14] aims to cope with this data deficit by pre-training a network on either non-agricultural open access data [9] or by generating synthetic data [14]. Both methods have shown promising results. An alternative direction explored in this paper is the development of algorithms based on smaller datasets, which can match the detection performance of machine learning algorithms and exceed their frame-rate—without the need for complex and expensive hardware (such as GPUs).

**Figure 1:**
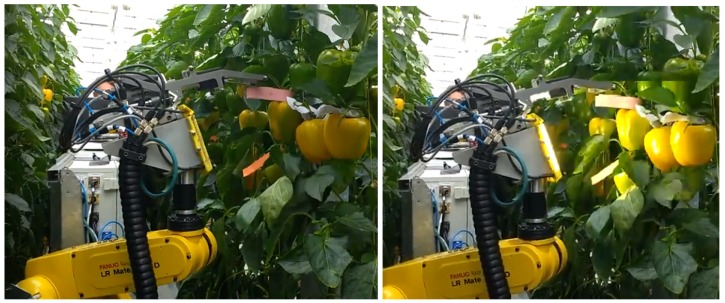
Experimentalsetting for FNF image acquisition; the same image is taken twice, with (**right**) and without (**left**) artificial light.

**Figure 2:**
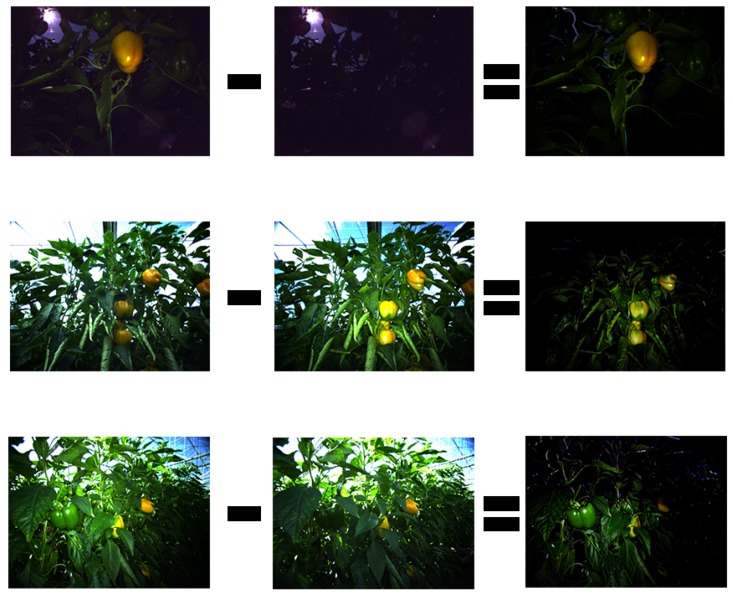
Examples of the Flash-no-Flash process images taken facing the sun (**top row**), facing away from the sun (**center row**), and facing to the side (**bottom row**). By subtracting the luminance values of the No-Flash image (**center column**) from the Flash-only image (**left column**), natural scene illumination is removed (**right column**).

For an algorithm to be practical in the robotics domain, it must remain efficient in terms of computational power. To ensure applicability and usability of a robotic harvester it must be easily adjustable to the highly variable conditions. The variability in the scene appearance that a harvesting application must process is caused by three main sources [11,13]: object variability, environment variability, and hardware variability. Object variability is a characteristic of its biological nature, in addition to the variation caused by the different growing and environmental conditions, resulting in differences in size, color, shape, location, and texture of the targets. Environment variability includes unstructured obstacle locations (e.g., leaves, branches) and changing lighting conditions (e.g., day/night time illumination, direct sunlight, shadows), which depend on time and location and directly affect the performance of the detection algorithms. The specific robotic system modules used (sensors, illumination, and manipulator design, including degrees of freedom, dimensions, and controls) also affect the image quality and thus influence detection performance. Therefore, segmentation algorithms developed for other domains (e.g., medical imaging [15,16], or sensing for navigation in indoor environments [17,18]) often fail in outdoor and agricultural domains [2,19,20,21]. To overcome such variable conditions, some parameters of the environment must be stabilized. In this paper we present the Flash-no-Flash (FNF) approach [22] to stabilize the impact of the ambient lighting conditions on the image. This controlled illumination acquisition protocol frees the system from most ambient illumination effects and facilitates robust target detection while using only modest computational resources and not requiring supervised training. This approach relies on the acquisition of two images in Figure 1 nearly simultaneously—one with a strong artificial light (“Flash”) and one with natural light only (“no-Flash”). The difference between the two images represents the appearance of the target scene as if only the artificial light was present (Figure 2). In order to maximize ambient light reduction, camera exposure was set to the lowest possible setting (20 μs). As can be seen in Figure 2, even these short exposures could not completely remove strong ambient light sources, such as direct sunlight. Furthermore, once ambient light has been removed by the FNF process, the artificial light source becomes the scene’s main illuminant. Since flash intensity is quickly reduced over distance, items closer to the camera remain properly exposed for detection while the background and the items further away from the camera remain dark and filtered out. This is specifically beneficial for robotic tasks within the greenhouse environment where adjacent crop rows are a significant source of visual confusion. These FNF composite images can then serve as the basis for a simple and robust color-based detection algorithm.

Alternatively, more complex algorithms have been used for the robust detection of fruit or vegetables using artificial neural networks [4,6,23], where the Faster Region-based Convolutional Neural Networks (Faster R-CNN) detector [24] was modified for fruit detection. Another recent network for object detection is Single Shot MultiBox Detector (SSD) [25], which was shown to provide accurate results and a faster runtime [26]. Such methods have been shown to provide accurate detection results; however, they may be difficult to train and require additional computational resources (i.e., a GPU), without which the computation may be rather slow. This typically requires a well-sized platform to host those resources, a limitation that may prohibit certain applications.

While the results of the proposed algorithms are in most cases better than the basic detection algorithm, the complexity of the advanced algorithms and their appetite for training data are major limiting factors for implementation in greenhouse conditions. In their recent review, Kamilaris et al. [6] mentioned that most deep-learning based algorithms to date have been trained and tested on data from the same greenhouse; therefore the transferability of the obtained results to different environmental conditions remains questionable. Both the advanced hardware and the need for fine-tuned training procedure may once again increase the attractiveness of simpler algorithms, especially when aimed for robotic applications that require fast detection. This paper aims to explore this issue in depth.

To analyze the proposed method (as is the case with any method), greenhouse data acquisition is required. Indeed, the evaluation of all possible variations in environment, object, and robotic properties requires acquisition of extensive datasets and thus should be automated. In this paper we present automated data acquisition with a robotic manipulator that implements acquisition protocols [13]. These datasets enable advancement of vision algorithms development [27] and provide a benchmark for evaluating new algorithms. To the best of our knowledge limited-size agricultural databases has been released (e.g., [4,28]. Table 1 includes summary results of number of images and fruit type that has been released to the public and documented in recent reviews [1,6]. Evaluation of previously reported color-based algorithms was based on earlier limited data but indicated the importance of evaluating algorithms for a wide range of sensory, crop, and environmental conditions [1]. The main contributions presented in this paper are: an automatic methodology for dataset acquisition, detection algorithms developed over the acquired dataset, and the dataset itself, which is publicly released for the benefit of the scientific community.

## 2. Algorithms

The FNF contribution to detection was evaluated by comparing its performance to a simple detection algorithm and an elaborate deep learning model, both on the Flash-only and FNF data.

### 2.1. FNF Algorithm

Due to the use of complex (custom illumination light-emitting diode array driven by an independent controller) experimental hardware (pre-production customized prototype of the Fotonic F80 camera)—implementation of the FNF procedure was not entirely straightforward and included the following steps:

- **Detect Flash/No-Flash Illumination**
  While the camera was configured to alternate triggering of the LED array between frames, various factors could interrupt this timing such as: the camera’s variable frame-rate, “dropped” frames, and communication latency between various system components (camera/PC, camera/LED controller). This necessitated constant evaluation of the incoming image stream in order to determine which images were taken under flash illumination. To accomplish this goal, the average brightness of consecutive images was compared, and if it exceeded a manually defined threshold the images were considered a valid FNF pair. The system’s FNF threshold was determined once via field-testing, and provided stable performance throughout the database acquisition process.
- **Subtract Latest Flash/No-Flash Image Pair**
  Once a valid FNF image pair was acquired, the ”no-Flash” image was subtracted from the “Flash” image on a per-pixel basis. Color artifacts were avoided by excluding overexposed or “saturated” pixels in the “Flash” from this subtraction process. Similarly, pixels that contained negative values following this process were corrected to 0 in order to produce a valid RGB image.
  The basic process of FNF image acquisition and its results are demonstrated in Figure 2.

### 2.2. Color-Based Detection Algorithm

**Figure 3:**
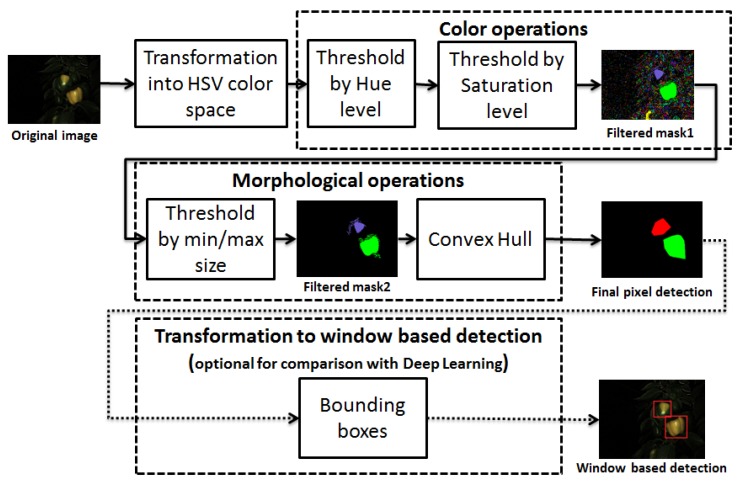
Color-based algorithm flowchart. The additional transformation from pixel-based detection to window-based detection is made for comparison with the deep learning algorithm.

This approach was selected to be as simple and as naive as possible, namely threshold-based detection (Figure 3) of the targets applied on the following features:

- Hue level: 20/360–50/360
- Saturation level: 90/255–255/255
- Minimum object size: 400 px (image resolution: 320 × 240)

The features thresholds were calibrated using Matlab’s “color thresholder” app, by processing 3 randomly sampled images. The app allows dynamic review of the image mask when applying various threshold levels on 3 of the defined color channels (H, S, and V). Each image was reviewed by a human operator to provide HSV thresholds that would best separate the fruits from the background. The measure for best separation was subjective and included the thresholds that would create no large area false positives with minimal removal of the area of the detected targets.

**Figure 4:**
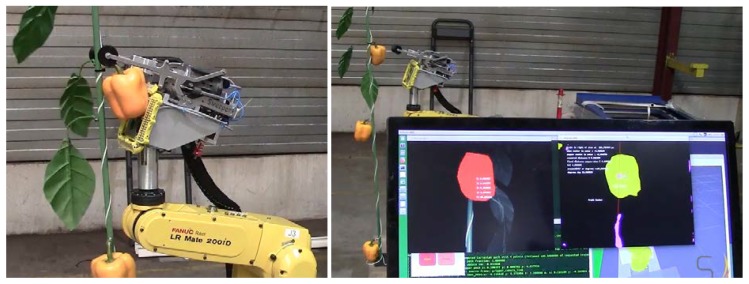
Detection of an artificial plant model via color-based detection after threshold re-calibration. Image courtesy Bogaerts Greenhouse Logistics. The robotic harvesting system successfully detects and “harvests” an artificial pepper fruit (**left**) while the detection algorithm’s results are displayed to the operator on a graphical user inteface (**left**).

This simple calibration approach was designed to require only a small number of “training” images (3) and can be performed quickly—thus facilitating rapid adaptation to new environments (e.g., different greenhouses, growing conditions, pepper varieties). This advantage was utilized during the SWEEPER pepper harvesting robot development [29] in order to adapt the algorithm to an artificial plant model used for indoor testing (see Figure 4).

### 2.3. Deep-Learning Based Algorithm

We adopted a neural network-based SSD detector [25] due to its high speed and accuracy. This detector is based on consequent convolutional layers that predict box locations without region pooling, providing a fast detector. To enable the detection of peppers, the size of the last layers was reduced to predict two object classes (pepper or background) and the learning rate reduce from 0.00004 to 0.000001. Apart from that, no additional tuning of hyper-parameters was required (Complete parameter information for the SSD detector can be found on the GitHub repository associated with the corresponding publication [25] (https://github.com/weiliu89/caffe/blob/ssd/examples/ssd/ssd_pascal.py).).

## 3. Methods

The following section describes the data acquisition methods, the databases, the data processing and labelling, and analysis methods (performance measures and sensitivity analysis).

### 3.1. Data

**Figure 5:**
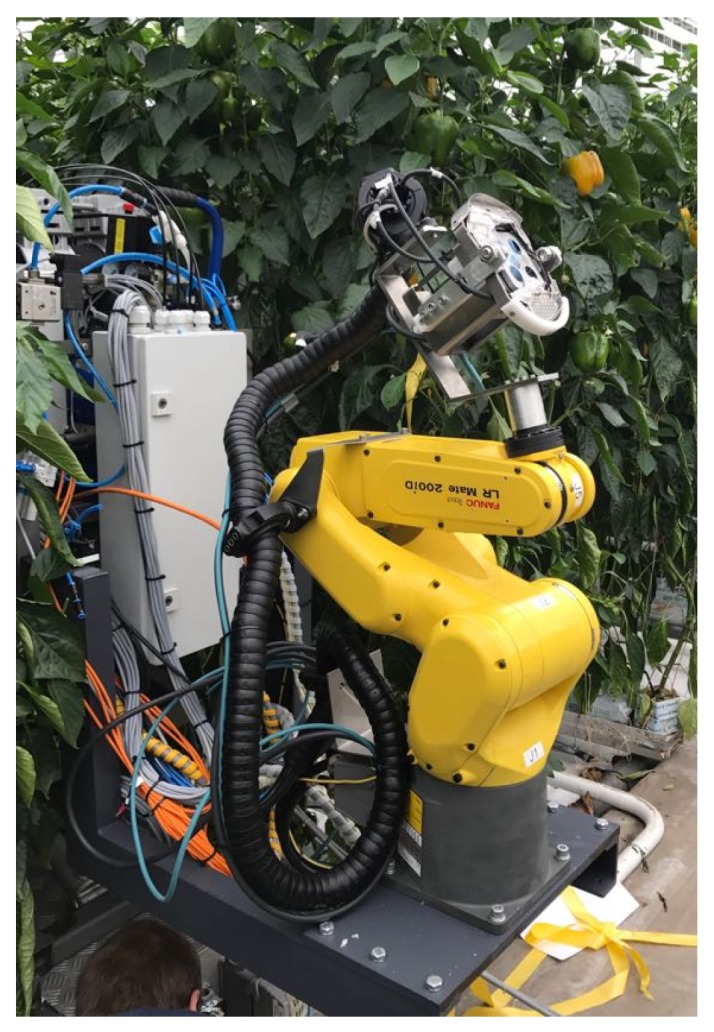
Experimental setup including a robotic arm, equipped with an RGBD camera and an illumination rig.

A database was acquired (http://icvl.cs.bgu.ac.il/lab_projects/agrovision/DB/Sweeper04/) in June 2017, during the 12th harvesting cycle in a commercial greenhouse in Ijsselmuiden, Netherlands. The pepper cultivar was Gualte (E20B.0132), seed company—Enza Zaden. Data was acquired in different natural lighting conditions (direct/indirect sunlight at various times of day, and various angles relative to the sun) along three consecutive days using the experimental setup described in Figure 5. These scenes incorporated peppers of all maturity classes (mature/non-mature/partially mature). The data collection experiment resulted with a total number of 168 scenes that included 344 peppers.

### 3.2. Data Acquisition

**Figure 6:**
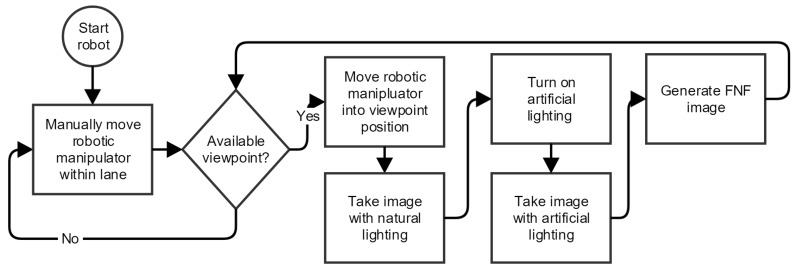
Data acquisition protocol.

The setup consists of a 6 degree of freedom industrial manipulator (Fanuc LR Mate 200iD), equipped with a Fotonic F80 camera (hybrid RGB-TOF depth camera capable of providing 320 × 240 RGB-D images at 20 fps, specially customized with an external illumination trigger) and a specially designed 3D-printed illumination rig (four custom-ordered Effilux brand LED strips, each containing two columns of 17 Osram LEDs, with a total of 136 LEDs) to automatically acquire RGB images and depth information from three viewpoints as described in Table 2 with both artificial and non-artificial illumination conditions (Kurtser et al., 2016). This acquisition was performed automatically according to the procedure described in Figure 6.

Acquired scenes were selected randomly within the row. To ensure the scenes include peppers, the robotic manipulator sensory system was placed manually in front of a pepper or a cluster of peppers in the scene before starting the automatic procedure. The scenes were acquired all along the day on both sides of the aisle to collect data with variable natural illumination conditions (e.g., against or in front of the sun).

### 3.3. Data Processing and Labelling

**Figure 7:**
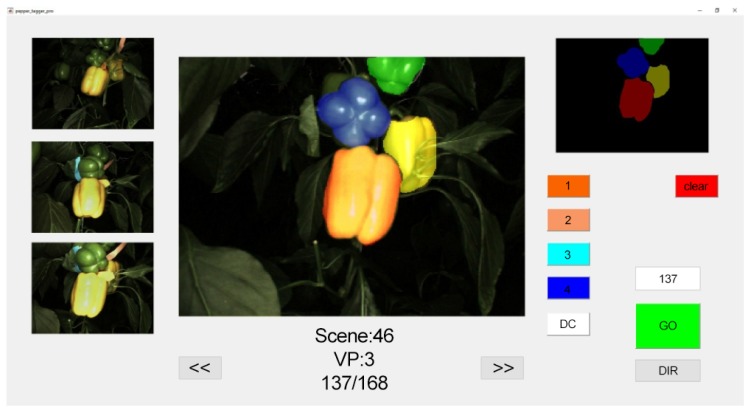
Custom-made user interface developed for labeling database images.

Since labelled data was necessary for both evaluation of performance and training of the deep network algorithm, a manual labelling process was conducted using a custom-made user interface designed and implemented in Matlab 2017a (Figure 7). Each image being labelled as well as the other 3 available viewpoints from the scene the image was taken from, were visible to the user. The user could classify the observed peppers into up to 4 classes marked in different colors. The resulting mask was then stored for future use.

### 3.4. Performance Measures

To evaluate performance the following measures were calculated:

- **FNF images vs. Flash-only images**. To evaluate the impact the FNF acquisition methodology has on the appearance of the processed images, we first computed the distribution of hue and saturation of images acquired with the FNF protocol and compared them to the same measures for the Flash-only images.
- **Detection accuracy measures**. To evaluate the detection rate provided by the algorithms we computed precision and recall (Equations (1) and (2)) performance of both algorithms on both the Flash-only and FNF data.
  (1) $$Precision=\frac{N_{TP}}{N_{TP}+N_{FP}}$$
  (2) $$Recall=\frac{N_{TP}}{N_{TP}+N_{FN}}$$
  where $N_{TP}$ is the number of correctly detected peppers (peppers were considered correctly detected if the bounding boxes of the detection and label have an overlap ratio (The overlap ratio of two bounding boxes is defined as the ratio between the area of their intersection and the area of their union.) of at least 50%)); $N_{FP}$ is the number of incorrectly detected peppers (where a detection was produced but its bounding box did not satisfy the overlap ratio criteria with any labeled pepper); $N_{FN}$ is the number of peppers that were tagged but had not been detected (where a detection was either not produced, or produced but failed to satisfy the overlap ratio criteria with the labeled pepper).
- **Time measures**. To evaluate the resources required for the color-based detection algorithm as opposed to the advanced deep learning algorithm, the training times and operation times were logged on different hardware.

**Figure 8:**
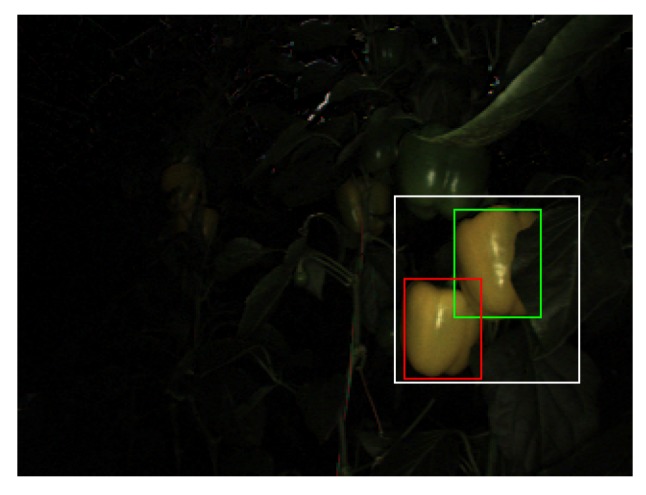
Example of a clustered detection: the detected area (**white**) overlaps both a pepper considered to be a true positive detection (**green**) as well as one considered a false negative detection (**red**).

Extra care should be provided for how clusters of peppers should be treated in the performance measures analysis. Figure 8 portrays an example of overlapping peppers: a cluster of two or more peppers was detected but due to morphological operations the cluster was identified as a single pepper. For robotic harvesting, detecting a cluster of peppers as a single fruit implies incorrect localization of the target pepper. Since most harvesters today are equipped with a visual servoing mechanism to approach the fruit [30,31], this error will be fixed either while approaching the fruit or after harvesting one of the fruits in the cluster.

There are two detection accuracy measures presented in this paper. The first measure considers each of the undetected peppers in a cluster as a false negative. The second measure considers each of the peppers within the detected area to be a true positive.

### 3.5. Sensitivity Analysis

The relation between the precision and recall might change along the performance of the robotic task [32]. In overview images taken by the robotic harvester it is important to maximize the TP rates and lower FP rates to ensure low cycle times (ensure the robot does not waste time on false targets). On the other hand, in visual servoing mode, after the target has been located the arm must then be guided accurately towards it for harvesting. At this stage, the detection task becomes “easier” since the target is centered and close to the camera. Here, reducing FP should be emphasized to avoid misguiding the arm, while the TP rate is less significant. Therefore, the relation between TP and FP as a function of the algorithm’s parameters is also analyzed.

The color-based detection algorithm was evaluated across the entire dataset for two evaluation schemes:

1. “Strict”—Detection of partially matured peppers considered a false positive.
2. “Flexible”—Detection of partially matured peppers considered a true positive.

It should be noted that since the detection algorithm is color-based, the chances of incorrectly detecting a partially mature pepper is directly proportional to the level of maturity of that pepper.

## 4. Results

### 4.1. FNF Images vs. Flash Only Images

**Figure 9:**
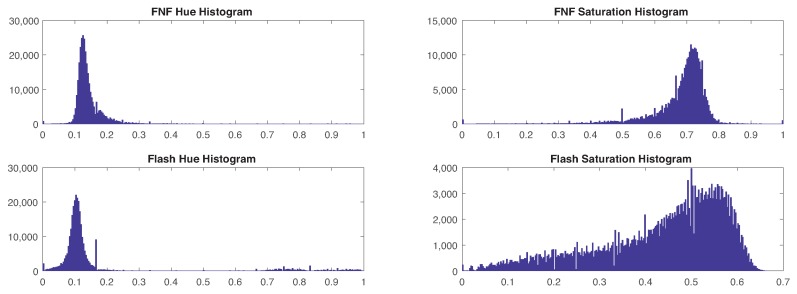
Histograms depicting the value distribution of hue and saturation for pepper pixels under FNF and Flash-only illuminations. From the top left, clockwise: FNF Hue histogram, FNF saturation histogram, Flash saturation histogram, and Flash hue histogram.

Studying all pixels labeled as ripe peppers under both FNF and Flash-only images revealed significant differences in the distribution of both hue and saturation. Histograms depicting the value distribution for both parameters can be found in Figure 9. The standard deviation for hue was 0.045 for FNF and 0.203 for Flash-only, and the standard deviation for saturation was 0.091 for FNF and 0.138 for Flash-only. This significant reduction of sample variance in FNF images vs. Flash-only supports the claim that FNF provides higher color constancy, thus facilitating better performance in color-based detection algorithms.

### 4.2. Color-Based Detection Results

**Figure 10:**
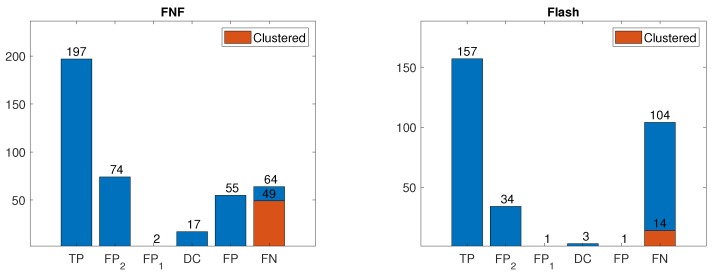
Distribution of detections for the color-based algorithm for FNF (**left**) and Flash-only (**right**) images. Proportion of false negatives due to clustering displayed in red.

Table 3 details the performance of the color-based detection algorithm under both “strict” and “flexible” evaluation schemes. Figure 10 details the distribution of detections, including an analysis of false positive cases:

- TP—Correct detection of a fruit.
- FP_2_—Partially-mature fruit detected as mature.
- FP_1_—Non-mature fruit detected as mature.
- DC—Distant, out of range, fruit detected (ignored).
- FP—False detection (no fruit at detected location).
- FN—False misdetection (fruit present but not detected)

False negatives due to pepper clusters were a significant proportion of false negatives in the FNF configuration (as evident from Figure 10). While such errors do reduce localization accuracy, they do not necessarily indicate a failure to harvest since the “undetected” pepper may be harvested once the detected pepper is harvested. Moreover, detected clusters are often separated into discrete detections as the robot arm approaches them during the harvesting procedure. Table 4 details the performance of the color-based detection algorithm, when clustered detections (cf. Figure 8) are considered successful. The color-based detection algorithm achieved a throughput of 30 fps when run on an Intel^©^ Core^TM^ i7-4700MQ 2.4 GHz CPU.

### 4.3. Deep Learning Results

**Figure 11:**
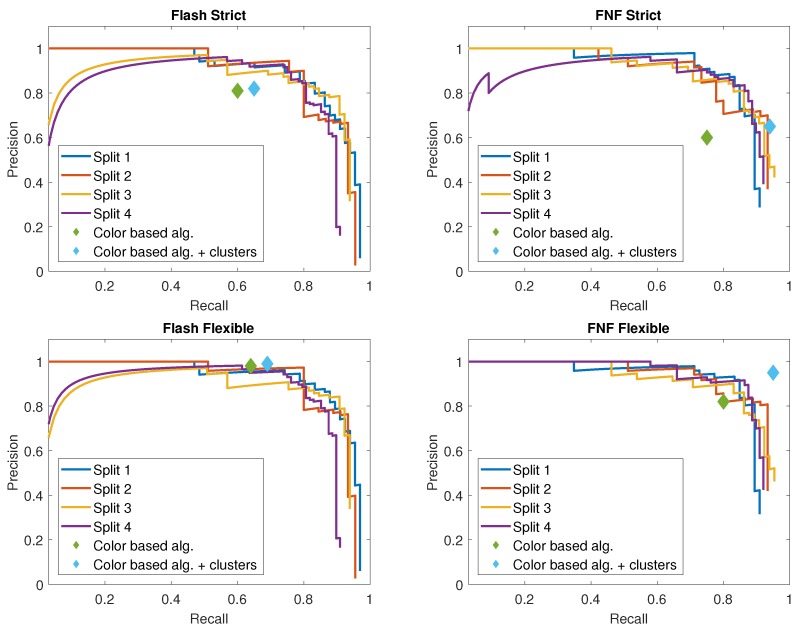
Precision-recall (PR) graph of the SSD object detection over each split of the Flash-only and FNF datasets. Color-based detection algorithm performance displayed for comparison. Figures depict: PR under the strict evaluation scheme for flash images (**top left**) and FNF images (**top right**), and PR under the flexible evaluation scheme for flash images (**bottom left**) and FNF images (**bottom right**).

The network is evaluated with the dataset described in Section 3.1, using 4 different train and test splits (see Table 5). We train and test separately on Flash-Only and FNF images, achieving an AP of (0.8341, 0.8478, 0.8482, 0.8326) over Flash splits, and (0.8493, 0.8601, 0.8437, 0.7935) over FNF splits, where the mean across splits is 0.8407 and 0.8367, respectively. In addition, precision-recall curves for all splits can be seen in Figure 11. While the effect of pepper clusters was quite pronounced in the color-based detection algorithm, the trained network was found to be robust to pepper clustering and did not detect large clusters as single peppers.

As can be seen, the neural network results are not significantly different over the two modalities. Reported results were achieved with an SSD network based on VGG16, operating at 30 images per second on Titan X GPU, or requiring a runtime of 3.5 s per image when run on an Intel^©^ Xeon^©^ Processor E5-2637v4 3.5GHz CPU.

These performance measures should be considered an approximate upper bound for the deep-learning approach since, as noted by Kamilaris et al. [6], performance may decrease or comprehensive retraining may be required if the operational environment changes (e.g., different greenhouses, growing conditions, lighting).

## 5. Conclusions

Analysis of ripe peppers’ hue and saturation distribution in Flash-only and FNF images revealed a significant reduction in variability for FNF images, suggesting higher color stability. This had, as expected, a positive effect on the performance of the color-based algorithm. The color-based algorithm was shown to obtain a maximum of 95% precision at a 95% recall level on FNF images compared to 99% precision at a 69% recall for Flash-only images. This result suggests FNF is a successful tool in stabilizing the effects of illumination for color-based detection algorithms. The detection results for deep learning techniques showed similar results for both Flash-only and FNF images (average precision 84% and 83.6%, respectively), implying that this approach can overcome variable illumination, without the FNF correction, at the cost of additional computation. The FNF color-based algorithm achieves comparable performance to the deep-learning approach despite its simpler methodology (cf. performance points in Table 3 and Table 4 as plotted on Figure 11).

Performance comparisons for both algorithms on a variety of server, desktop, and embedded platforms (cf. Table 6) revealed that the color-based algorithm offers high performance in low-cost embedded systems requiring real-time continuous detection, while the deep learning algorithm requires specialized and costly hardware to perform in real-time. These results imply that while the simpler algorithm suggested is indeed naive and may under-perform the advanced machine-learning algorithm in some settings, it remains an appealing alternative for real-time embedded systems that cannot afford the use of an on-board GPU in the field. In such cases, the FNF approach may provide better overall performance due to its higher frame-rate and allow acquisition of multiple viewpoints [13], increasing detectability and enabling visual servoing continued re-detection, a common practice in robotic harvesting [30].

Other color-based algorithms could benefit from the implementation of FNF imaging as well, and can be tested and benchmarked over the published FNF dataset.

## Figures and Tables

**Table 1:** Summary of previously published results and comparison to proposed methods.

Paper	Crop	Dataset	Algo.	FPR	TPR	F	A	P	R
Ostovar et al., 2018 [7]	Sweet peppers	170 img	AD	–	–	–	91.5%	–	–
Chen et al., 2017 [8]	Apples	1749 (21 img)	DL	5.1%	95.7%	–	–	–	–
	Oranges	7200 (71 img)		3.3%	96.1%				
McCool et al., 2017 [9]	Weed	Pre-train: $10^{6}$ img tune & test: 60 img	D-CNN	–	–	–	93.9%	–	–
Milioto et al., 2017 [10]	Weed	5696 (867 img) 26,163 (1102 img)	CNN	–	–	–	96.8% 99.7%	97.3% 96.1%	98.1% 96.3%
Sa et al., 2016 [4]	Sweet pepper	122 img	DL	–	–	82.8%	–	–	–
	Rock melon	135 img				84.8%			
	Apple	64 img				93.8%			
	Avocado	54 img				93.2%			
	Mango	170 img				94.2%			
	Orange	57 img				91.5%			
Vitzrabin et al., 2016 [11]	Sweet pepper	479 (221 img)	AD	4.6%	90.0%	–	–	–	–
Zheng et al., 2009 [12]	Vegetation	20 img 80 img	Mean-Shift	–	–	–	95.4% 95.9%	–	–
Our Results (FNF strict/flexible)	Sweet pepper	156 img	AD	–	–	–	–	65%/95%	94%/95%
Our Results (SSD)	Sweet pepper	156 img	DL	–	–	–	–	84%	–

DL = Deep learning; AD = Adaptive threshold; F = F${}_{1}$ Score; A = Accuracy; P = Precision; R = Recall.

**Table 2:** Viewpoints description.

View Point	Distance to Stem (mm)	Tilt (Degrees)	Azimuth (Degrees)
**1**	190	10	−50
**2**	190	20	20
**3**	170	0	0

**Table 3:** Performance evaluation of the color-based algorithm across the entire data-set.

Image Type	Measure	Strict	Flexible
FNF	Recall	75%	80%
	Precision	60%	82%
Flash-only	Recall	60%	64%
	Precision	81%	98%

**Table 4:** Performance evaluation of the color-based algorithm across the entire dataset, when pepper clusters are considered successful detections.

Image Type	Measure	Strict	Flexible
FNF	Recall	94%	95%
	Precision	65%	95%
Flash-only	Recall	65%	69%
	Precision	82%	99%

**Table 5:** Image counts for train/test sets used to train and evaluate the deep-learning based algorithm.

	Train	Test
Split 1	128	40
Split 2	138	30
Split 3	129	39
Split 4	119	49

**Table 6:** Performance estimates for deep-learning and color-based detection algorithms. Performance predictions were extrapolated based on the core-count and clock-speed of target systems relative to measured performance on test systems (denoted in bold). Real world performance may vary due to the various hardware instruction optimization and parallelization capabilities of each platform. Note the color-based algorithm’s ability to provide high frame-rates on low-cost hardware embedded platforms such as the Raspberry Pi.

CPU	GPU	Approximate	Deep Learning	Color-Based
		System Cost	Performance	Performance
2 X Intel^©^ Xeon^©^ E5-2637v4 3.5 GHz	Nvidia Titan X	$9200	**30 fps**	44 fps
2 X Intel^©^ Xeon^©^ E5-2637v4 3.5 GHz	none	$7800	**0.28 fps**	44 fps
8-core ARM v8.2 64-bit CPU	512-core Volta GPU	$1400	33 fps	56 fps
Intel^©^ Core^TM^ i7-4700MQ 2.4 GHz	none	$800	0.19 fps	**30 fps**
Cortex-A53 64-bit SoC 1.4 GHz	none	$35	0.22 fps	35 fps
(Rasberry Pi 3 B+)				

$ = United States Dollars.
